# Maintenance Therapy in First-Line Gastric and Gastroesophageal Junction Adenocarcinoma: A Retrospective Analysis

**DOI:** 10.3389/fonc.2021.641044

**Published:** 2021-09-10

**Authors:** Daniel Walden, Mohamad Bassam Sonbol, Skye Buckner Petty, Harry H. Yoon, Mitesh Borad, Tanios S. Bekaii-Saab, Daniel H. Ahn

**Affiliations:** ^1^Department of Medical Oncology, Mayo Clinic Cancer Center, Phoenix, AZ, United States; ^2^Department of Medical Oncology, Mayo Clinic Cancer Center, Rochester, MN, United States

**Keywords:** gastric, esophageal, maintenance therapy, continuous therapy, fluorouracil and oxaliplatin treatment

## Abstract

**Background:**

Fluoropyrimidine with platinum-based chemotherapy has become the standard of care for advanced gastric and gastroesophageal (GEJ) cancer. Trials in colon cancer show that induction chemotherapy followed by maintenance chemotherapy is an efficacious strategy to maximize clinical response while minimizing toxicity. The current retrospective study aims to evaluate the efficacy and tolerability of maintenance versus continuous treatment in advanced GEJ malignancy.

**Methods:**

A retrospective analysis of patients with metastatic gastric/GEJ adenocarcinoma treated with fluoropyrimidine and platinum chemotherapy between 2007-2017 was performed. Patients who achieved at least stable disease after initial induction treatment were included. After 16 weeks of induction chemotherapy, patients were categorized into the continuous group if induction chemotherapy was continued and the maintenance group if chemotherapy was switched to maintenance fluoropyrimidine monotherapy or observed off treatment. Endpoints were progression-free survival (PFS), overall survival (OS), and toxicities.

**Results:**

In total, 90 patients met the criteria, 48 received continuous therapy, and 42 received maintenance. Baseline characteristics were comparable. No difference in PFS (9.9 *vs* 8.4 months p = .28) or in OS (16.1 *vs* 21.3 months p = .75) was observed, including after controlling for the best response on induction therapy and other variables. In patients on continuous induction therapy, there was a higher prevalence of grade three neuropathy (42.6% *vs* 9.8% p = .001) and neutropenic fever (13% *vs* 0% p =.03).

**Conclusions:**

Maintenance therapy following induction fluoropyrimidine and platinum-based therapy is associated with an improved toxicity profile and appears to have comparable efficacy to continuous treatment in metastatic gastric/GEJ cancer.

## Introduction

Gastric and gastroesophageal junction (GEJ) cancers remain one of the most prevalent malignancies globally and contribute to the second most common cause of cancer-related deaths in the world with over 750,000 deaths annually (1). Over 40% of gastric cancer diagnoses are made at an advanced stage (2). Combination chemotherapy with fluoropyrimidine and platinum-based therapies is the mainstay of treatment in patients with advanced-stage gastric/GEJ neoplasms (3–5). The identification and utilization of targeted therapies, including trastuzumab for HER-2 positive adenocarcinomas, have largely been adopted into clinical practice (6). However, with the small proportion of patients with adequate histological positive HER-2 status that would benefit from the addition of trastuzumab to cytotoxic therapy (7), most patients with advanced gastric and GEJ cancer would be limited to cytotoxic chemotherapy. Given the associated toxicities from prolonged exposure to cytotoxic chemotherapy, studies have been performed in metastatic colorectal cancer (mCRC) investigating various treatment schedules to optimize efficacy with minimizing toxicity (8–10). This has led to the adoption of maintenance strategy or observation as options after a period of induction chemotherapy in mCRC with less toxicity while preserving overall survival (11–13).

Similarly, in an attempt to decrease the side effects seen with platinum-based therapies in metastatic gastric/GEJ cancers, the question of maintenance therapy remains unanswered with only one study evaluating the feasibility of such an approach with S-1 maintenance in metastatic gastric adenocarcinoma (14). Based on the paucity of data, we evaluated the safety and efficacy outcomes of maintenance/observation (MTC/OBS) strategy compared to continuation of cytotoxic chemotherapy (CTX) in patients with metastatic gastric/GEJ cancer.

## Methods

We performed a three-site retrospective analysis of patients with metastatic gastric/GEJ adenocarcinoma treated with fluoropyrimidine and platinum-based chemotherapies at Mayo Clinic (Arizona, Florida, and Minnesota) between 2007 and 2017. Inclusion criteria included metastatic gastric or GEJ cancer patients who achieved at least stable disease after initial induction treatment with Eastern Cooperative Oncology Group (ECOG) performance status less than or equal to 2. Stable disease was defined per RECIST criteria version 1.1 (15). Patients were categorized into the “continuous group” (CTX) if they were continued on the full cytotoxic regimen beyond 16 weeks of therapy and patients were assigned to the “maintenance group/observation (MTC/OBS) if they received a maximum of 16 weeks of combined induction therapy and subsequently placed on single-agent fluoropyrimidine therapy or observed off treatment. Data were extracted from the medical record to determine progression-free survival, overall survival, and associated toxicities. Overall survival (OS) was measured from the date of initiation of chemotherapy to the date of death of any cause. Progression-free survival was measured from the date of initiation of chemotherapy to the date of disease progression or death. Patients were censored if there was no progression or lost to follow-up. Drug toxicities and their associated grading were quantified according to Common Terminology Criteria for Adverse Events (CTCAE) version 5.0. Pearson’s Chi-squared tests, One-way ANOVA models, and Cochran-Armitage test for trends were applied to ascertain significance between the two groups for categorical, continuous, and ordinal variables respectively. Poisson regression models with robust sandwich estimators were used to estimate the relative risks of binary toxicity indications. Kaplan-Meier method was used to estimate survival curves, and log-rank tests were used to compare them. Cox-proportional hazards models were applied to estimate hazard ratios. Inverse probability weighting was implemented in the Cox proportional hazards models, and the Poisson regression models to adjust for age, sex, chemotherapy regimen, and recurrence. Our level of statistical significance was p <.05.

## Results

A total of 90 patients met the inclusion criteria with 48 in the CTX and 42 in the MTC/OBS group. Baseline patient characteristics are listed in **Table 1**. In total, 14 of the 42 MTC/OBS were observed off treatment with the intent of re-introduction of therapy upon disease progression and were included in the MTC/OBS group. Overall, baseline characteristics were comparable between the two groups except that more patients in the MTC/OBS group achieving complete response after induction chemotherapy and there were no significant differences in the type of induction chemotherapy (**Table 1**). HER2 testing was notably positive in (n =19) 21.1% of patients and trastuzumab was added to the chemotherapy backbone. The median number of total administered cycles for the continuous group was 14.1 cycles (range: 9-49). The average number of total cycles for the maintenance group was 6.4 cycles (range: 2-8). Doublet regimens were the most commonly used in the induction period with) 70% (63/90) of patients receiving FOLFOX and 5.5% (5/90) receiving capecitabine plus oxaliplatin (CAPEOX).

**Table 1:** Patient characteristics and prognostic variables in maintenance and continuous groups.

Characteristics	Continuous (n = 48)	Maintenance (n = 42)
Gender		
Male	35 (72.9%)	33 (78.6%)
Age at diagnosis		
Mean	58.1	61.1
Range	23-79	22-81
Age >65		
Yes	18 (37.5%)	15 (35.7%)
Metastatic or Recurrent Metastatic		
Metastatic at Presentation	46 (95.8%)	37 (88.1%)
Recurrent Gastric/GEJ Metastatic	2 (4.2%)	5 (11.9%)
Best tumor response after induction chemotherapy		
Complete Response	0 (0%)	8 (19.0%)
Partial Response	33 (68.8%)	21 (50%)
Stable Disease	15 (31.2%)	13 (31%)
Sites of Metastasis		
Liver	32 (66.7%)	22 (59.5%)
Bone	9 (18.8%)	11 (26.2%)
Peritoneal	24 (50.0%)	23 (54.8%)
Adrenal	6 (12.5%)	3 (7.1%)
Ovarian	2 (4.2%)	1 (2.4%)
Brain	5 (10.4%)	7 (16.7%)
Local Lymph	43 (89.6%)	37 (88.1%)
Mediastinal Lymph	4 (8.3%)	7 (16.7%)
Lung	9 (18.8%)	9 (21.4%)
Malignant pleural effusion	5 (10.4%)	8 (19.0%)
Malignant ascites	14 (29.2%)	12 (28.6%)
Metastatic Sites >3		
Yes	26 (54.2%)	23 (54.8%)
Chemotherapy Regimen		
DCF	1 (2.1%)	2 (4.8%)
DOF	1 (2.1%)	1 (2.4%)
ECC	0 (0%)	1 (2.4%)
EOC	5 (10.4%)	7 (16.6%)
EOF	1 (2.1%)	1 (2.4%)
FOLFOX	32 (66.7%)	13 (31.0%)
FOLFOX + Trastuzumab	7 (14.6%)	11 (26.2%)
CAPEOX	1 (2.1%)	3 (7.1%)
CAPEOX + Trastuzumab	0 (0%)	1 (2.4%)

DCF, docetaxel, cisplatin; 5-flurouracil (5-FU); DOF, docetaxel, oxaliplatin, 5-FU; ECC, Epirubicin, cisplatin, capecitabine. EOC, Epirubicin, oxaliplatin, capecitabine; EOF, Epirubicin, oxaliplatin, 5-FU; FOLFOX, 5-FU, leucoverin, oxaliplatin; CAPEOX, Capecitabin, oxaliplatin.

**Figure 1:**
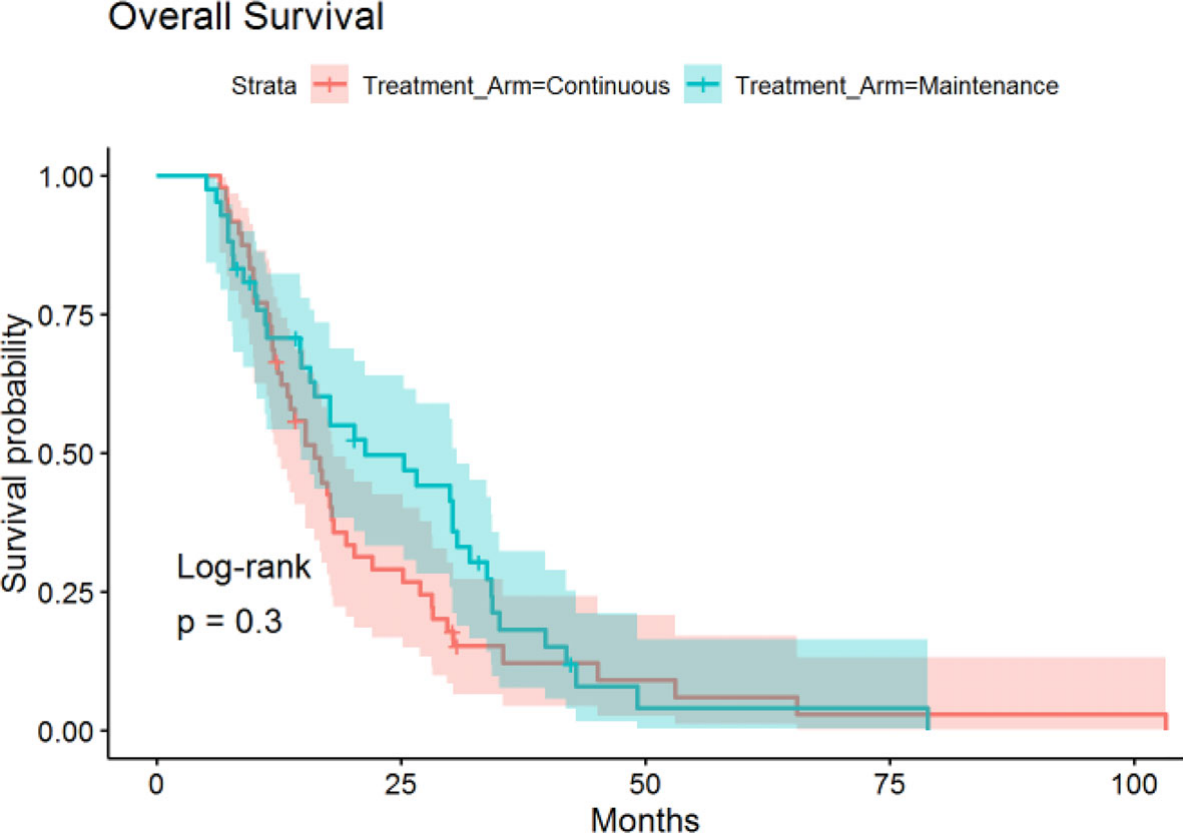
Overall Survival in Continuous *vs* Maintenance Groups.

**Figure 2:**
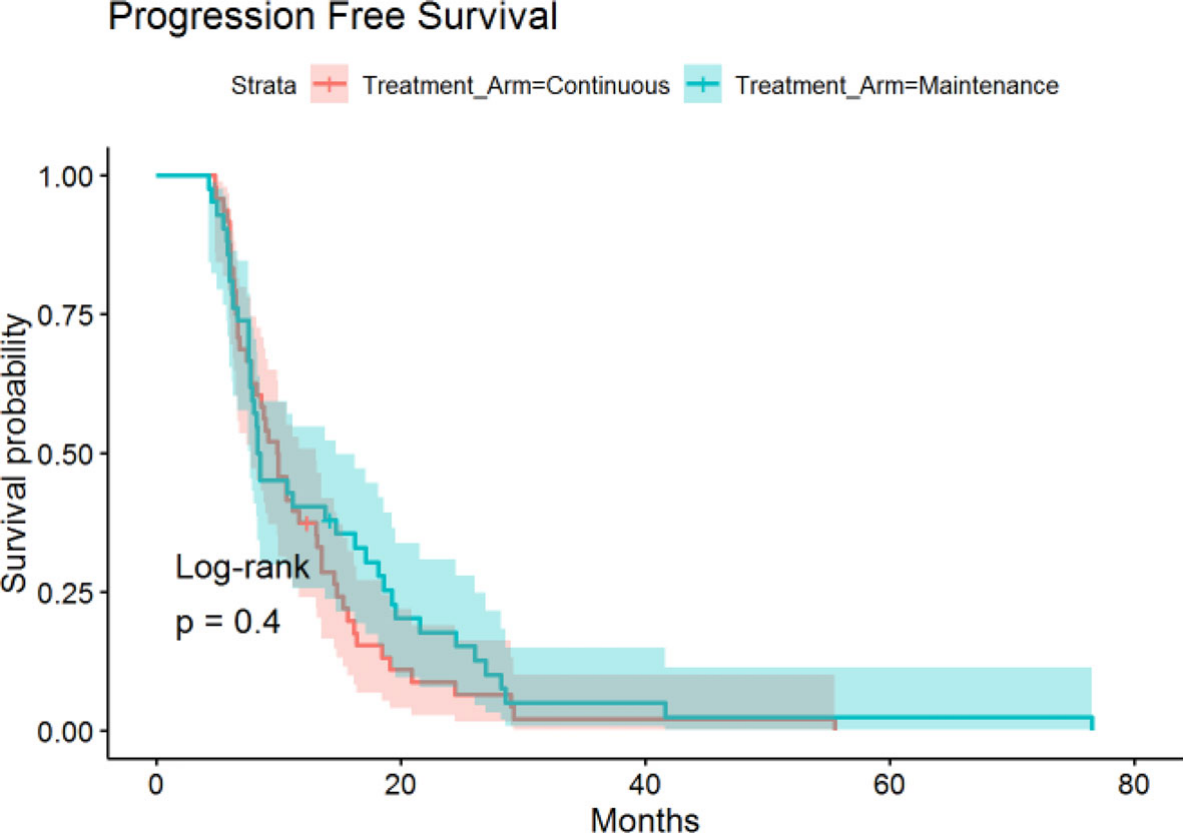
Progression-Free Survival in Continuous *vs* Maintenance Groups.

The median time of follow-up was 16.4 months. PFS was not significantly different between the continuous or maintenance groups (median = 9.9 *vs* 8.4 months, p = .28, HR=.86, 95% CI:.56-1.32) (**Figure 1**), nor was OS (median = 16.1 *vs* 21.3 months, p = .75, HR=.81, 95% CI:.51-1.28) (**Figure 2**). Grade 3 neuropathy was more prevalent in patients who received continuous therapy when compared to the maintenance group (42.6% *vs* 9.8% p =.001, RR = .21, CI:.07-.60). Additionally, patients who received continuous chemotherapy were associated with a significantly higher incidence of neutropenic fever (13.0% *vs* 0% p =.03). However, the development of grade 3 or 4 neutropenia was similar between the two groups (26.1% *vs* 37.5% p = .36). Other side effects, including anemia, thrombocytopenia, and any other grade 3 toxicity, were not significantly different between the continuous and maintenance groups (**Table 2**).

**Table 2:** Toxicity Profile of Continuous and Maintenance Regimens.

Side Effect	Continuous (N = 48)	Maintenance (N = 42)	p- value
Anemia			
Average Baseline Hemoglobin	12.66	12.25	p = .30
Greatest Decrease in Hemoglobin	3.07	2.48	p = .14
Range in Decrease in Hemoglobin	0 - 8	0 - 6.2	
Grade 1 Anemia	15 (32.6%)	17 (48.6%)	p = .14
Grade 2 Anemia	23 (50%)	13 (37.1%)	
Grade 3 Anemia	8 (17.4.2%)	4 (11.4%)	
Neutropenia			
Average Baseline Absolute Neutrophil Count	4.52	4.73	p = .54
Greatest Decrease in ANC (SD)	2.96 (1.57)	2.82 (1.46)	p = .72
Range in Decrease in Neutrophils	0 - 7.5	0 - 5.44	
Absolute Neutropenia (ANC < 1500)	31 (67.4%)	20 (60.6%)	
Grade 3/4 Neutropenia	12 (26.1%)	12 (37.5%)	p = .36
Neutropenic Fever	6 (13%)	0 (0.0%)	p = .03
Growth Factor (Neupogen/Neulasata) Support	10 (21.7%)	6 (17.6%)	p = .97
Thrombocytopenia			
Platelet count < 150,000	39 (83.0%)	18 (50.0%)	p= .20
Neuropathy			p = .0003
Neuropathy Grade 1	2 (4.3%)	7 (17.1%)	
Neuropathy Grade 2	25 (53.2%)	30 (73.2%)	
Neuropathy Grade 3	20 (42.6%)	4 (9.8%)	
Any Grade 3/4 Toxicity	31 (64.6%)	20 (50%)	p = .29

Hgb, Hemoglobin; ANC, Absolute Neutrophil Count; SD, Standard Deviation.

**Figure 3:**
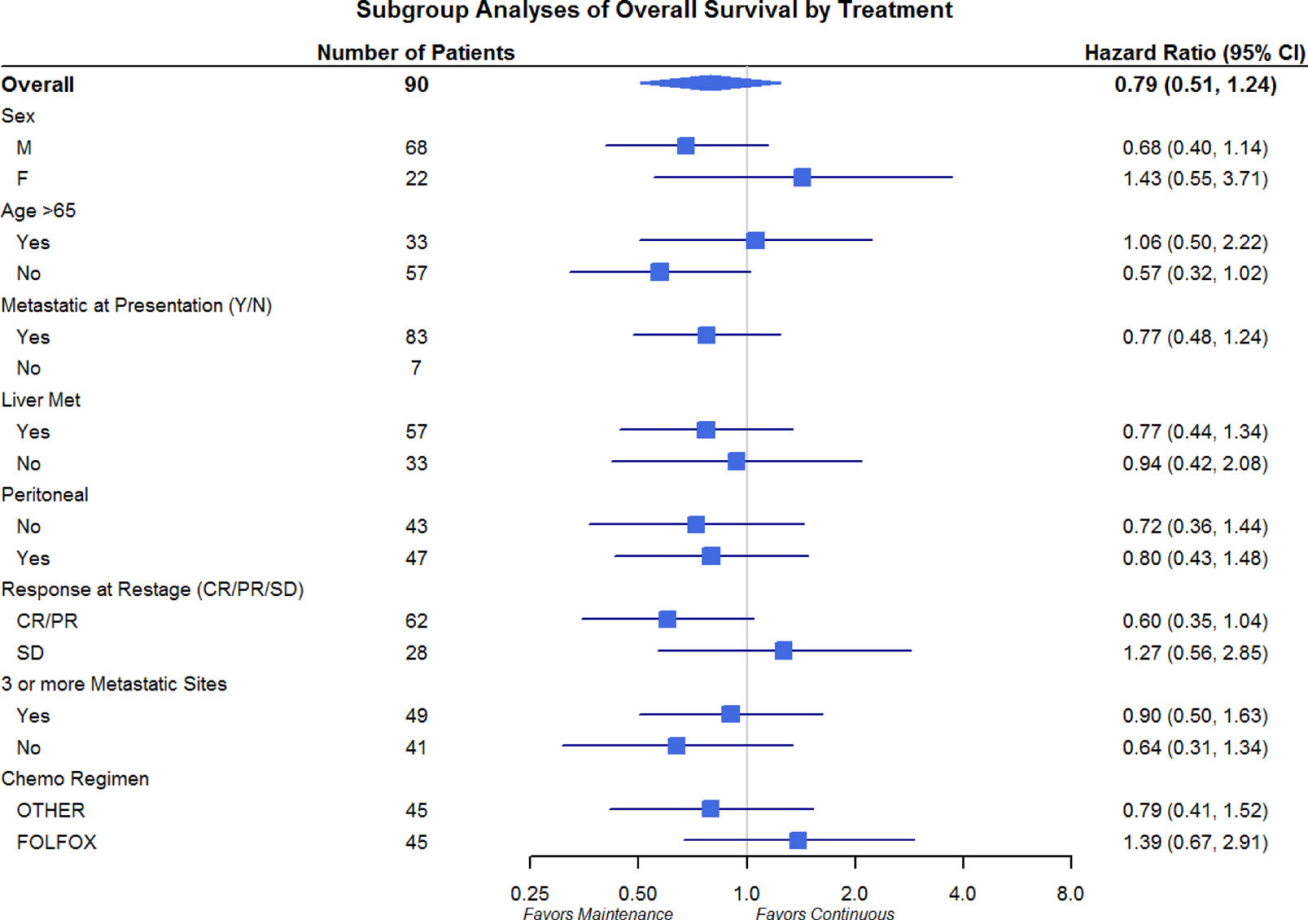
Overall Survival Subgroup Analysis of Gender, Age, Metastatic Site, and Chemo Regimen.

**Figure 4:**
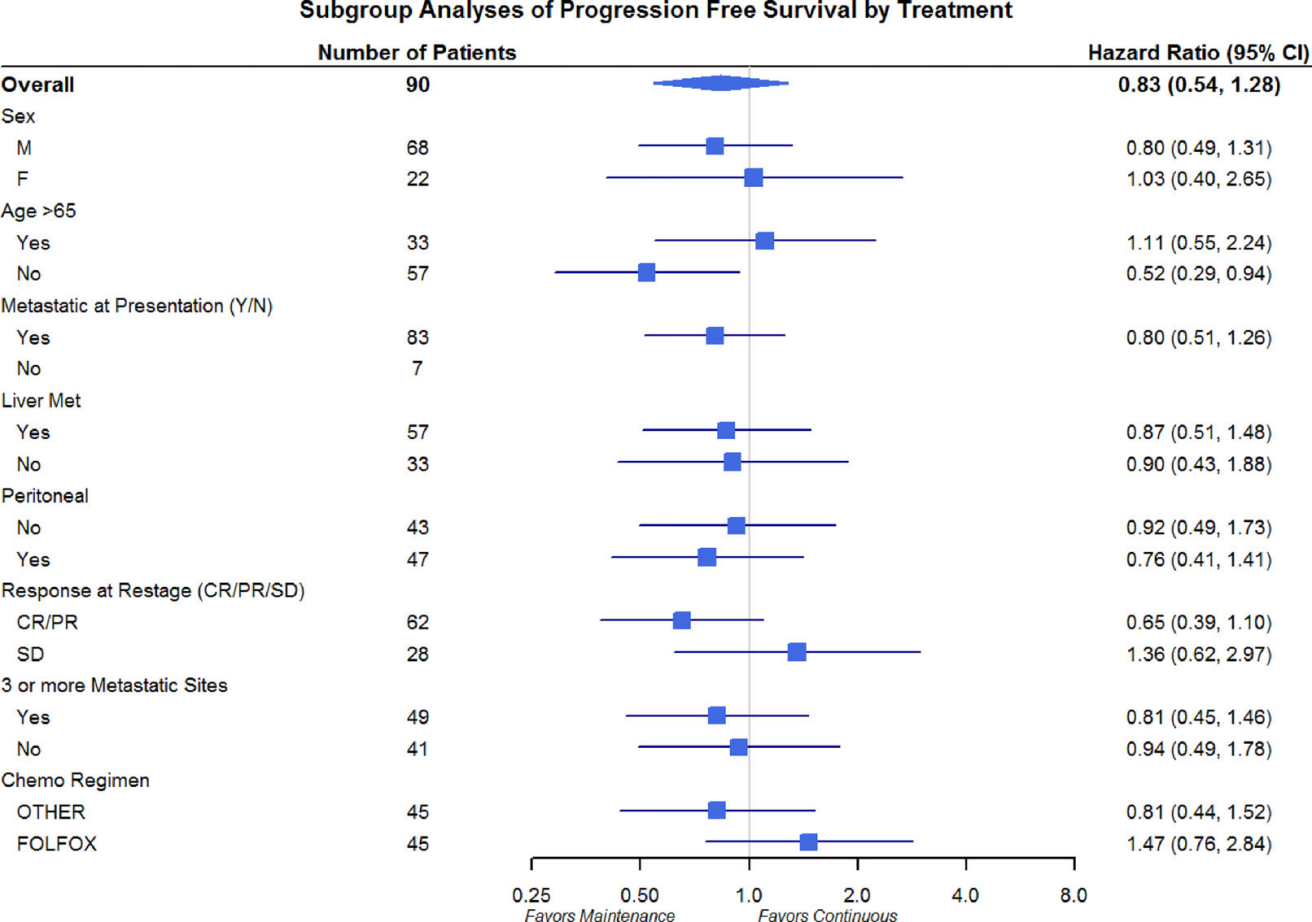
Progression-Free Survival Subgroup Analysis of Gender, Age, Metastatic Site, and Chemo Regimen.

**Figure 5:**
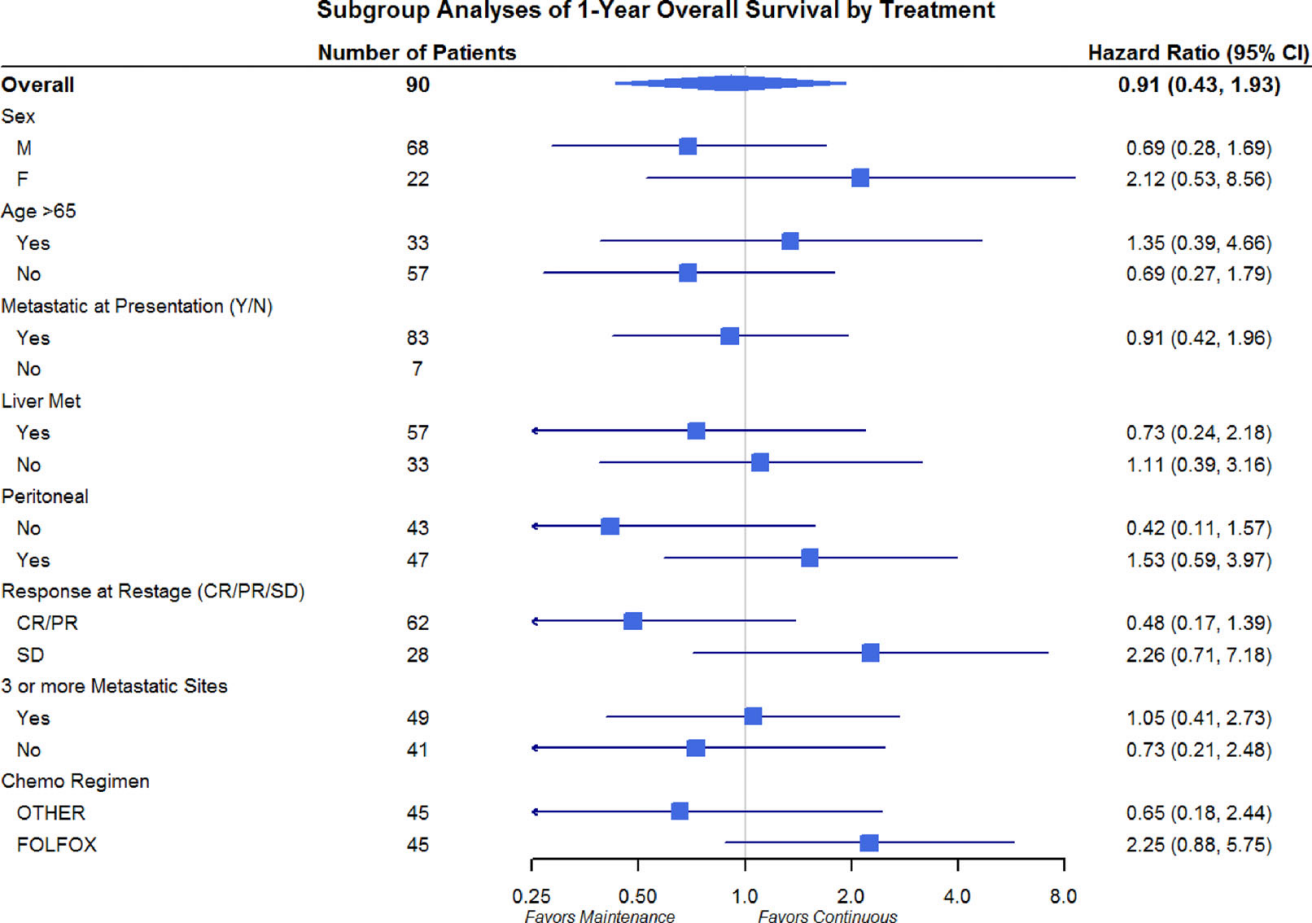
Overall Survival Subgroup Analysis of at 1 year: Gender, Age, Metastatic Site, and Chemo Regimen.

**Figure 6:**
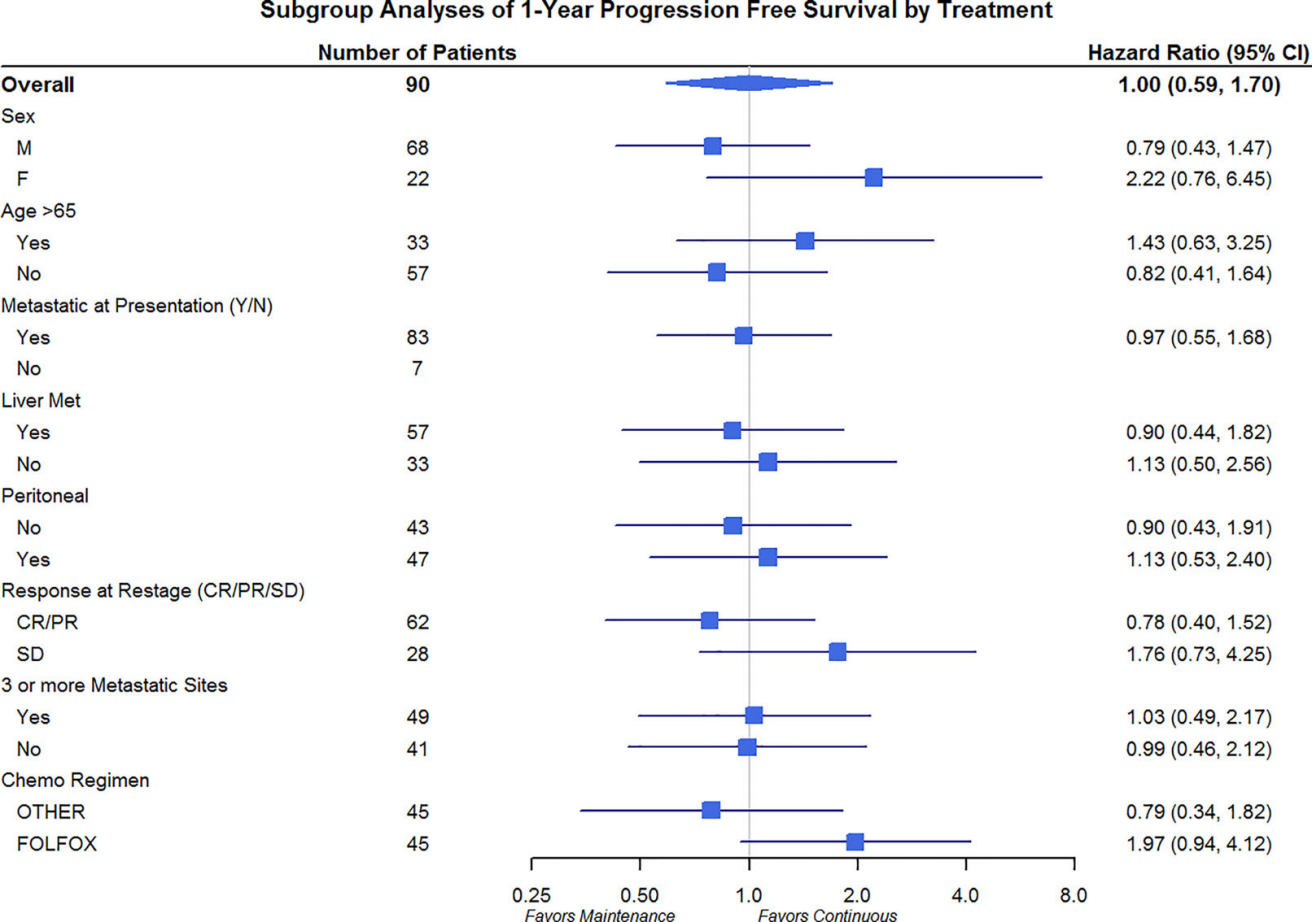
Progression-Free Survival Subgroup Analysis at 1 year: Gender, Age, Metastatic Site, and Chemo Regimen.

Differences in OS and PFS between continuous and maintenance therapy were examined within strata of potentially prognostic variables, such as gender, age, number of sites of metastasis, sites of metastasis, and chemotherapy regimen utilized. No significant differences were noted in the subgroup analysis in regards to PFS or OS (**Figures 3** and **4**). Similar findings were noted in a subgroup analysis of 1-year overall survival and progression-free survival (**Figures 5** and **6**).

## Discussion

The doublet combination of fluoropyrimidine and platinum chemotherapy has been the standard treatment in patients with advanced gastric or GEJ cancers (3, 4). Although the combination provides a significant improvement in survival, its administration is associated with significant toxicities, including peripheral neuropathy, cytopenias, and hepatotoxicities. Neuropathy is considered one of the most clinically significant side effects of platinum-based therapies and often a limiting aspect of continuing induction therapy (16, 17). Moreover, the associated cumulative toxicities and their effect on a patient’s performance status may limit effective administration of subsequent lines of therapy.

Randomized clinical trials in colorectal cancer have shown that continuation of induction therapy until progression is not superior to maintenance strategy in terms of PFS or OS (9, 11, 13), resulting in its adoption into standard practice. Furthermore, these trials suggest maintenance therapy produces less associated neuropathy when compared to continuous treatment. Currently, there is substantial variation in post-induction chemotherapy practice, though this is likely secondary to the lack of available studies in the field of metastatic gastric/GEJ cancer. The above knowledge provided the rationale to investigate the clinical efficacy and its toxicity profile for patients who received continuous induction chemotherapy compared to those who received treatment in a maintenance approach.

Our findings showed no significant differences in progression-free survival or overall survival in patients who switched to maintenance therapy or observation after a period of induction chemotherapy compared to continuous induction chemotherapy in patients with metastatic gastric/GEJ cancer. Furthermore, patients that were switched to maintenance treatment were associated with less peripheral neuropathy.

To our knowledge, besides our retrospective study, only one prospective study has evaluated the efficacy of maintenance treatment with a “stop-and-go” approach in gastric cancer (14). The study by Park et al. compared a “stop-and-go” approach with maintenance S-1 (oral fluoropyrimidine) to continuous induction therapy in terms of PFS and OS outcomes after six cycles of induction therapy (S1 with oxaliplatin) in metastatic gastric cancer. Although patients assigned to the continuous therapy arm had longer PFS (10.5 *vs* 7.2 months; HR 0.55 95% CI, .37-.81, p =.002) compared to the maintenance arm, there was no difference in OS 22.6 *vs*. 22.7 months, HR 0.78; 95% CI, 0.50-1.23; p = 0.284). In addition, consistent with our study, there were substantially more adverse events in the continuous arm compared to the maintenance arm with grade 3 fatigue (18.8% *vs* 8.1%) and sensory neuropathy (25.4% *vs* 9.7%). These findings were also noted in our study with grade 3 neuropathy (42.6% *vs* 9.8%) being more prominent in the continuous arm compared to the maintenance arm. This is especially important as the goal of therapy in advanced or metastatic disease is palliative with prolonging life while preserving quality of life. Additionally, overall survival was comparable between the “stop-and go” regimen (22.7 months) utilized in Park et al. study and the maintenance group in our cohort (21.3 months).

Ongoing studies investigating alternative agents as maintenance therapy for a patient whose progress in terms of induction therapy are currently underway. A recent prospective randomized clinical trial did not show added benefit maintenance therapy with the anti-PD-L1 antibody, avelumab compared to continuous chemotherapy until progression (18). While the toxicities were less in the avelumab group, the study was not powered for non-inferiority and the control arm was not maintenance chemotherapy. Additional trials are warranted for patients who progress on induction therapy, given their likely highly aggressive phenotype.

Overall the results of the current study, in conjunction with the results observed in Park et al. study, suggest that patients who are able to tolerate induction therapy and achieve at least stable disease may be better suited to receive single-agent fluoropyrimidine maintenance therapy or be observed off treatment with re-introduction with disease onset. A limiting aspect of the “stop-and-go” approach is whether patients will be able to tolerate additional platinum-based therapies upon disease progression. In the Park study, 37% of patients had to discontinue oxaliplatin after a median of 10 cycles due to neuropathy, neutropenia, or other adverse events suggesting that many patients will be intolerant of the resumption of dual therapy after disease progression. Similar findings were noted in the current study with 20% of patients having to discontinue platinum-based treatment due to neuropathy.

Limitations to the current study include the retrospective nature of the investigation with selection bias and associated confounding variables. Inverse probability weighting to the COX proportional hazard models was implemented in an attempt to adjust for confounding variables such as age, sex, and chemotherapy regimen utilized. Additional limitations include that the outcome data of many patients are from the pre-immunotherapy era, as the study covered patients treated from 2007-2017. Given these findings, and in an attempt to mitigate these aforementioned limitations, additional prospective, randomized clinical trials are warranted to further delineate the role of single-agent fluoropyrimidine maintenance therapy following induction chemotherapy in metastatic gastric cancer.

## Data Availability Statement

The original contributions presented in the study are included in the article/supplementary material. Further inquiries can be directed to the corresponding author.

## Author Contributions 

All authors contributed equally to the creation of this article. All authors contributed to the article and approved the submitted version.

## Conflict of Interest

The authors declare that the research was conducted in the absence of any commercial or financial relationships that could be construed as a potential conflict of interest.

## Publisher’s Note

All claims expressed in this article are solely those of the authors and do not necessarily represent those of their affiliated organizations, or those of the publisher, the editors and the reviewers. Any product that may be evaluated in this article, or claim that may be made by its manufacturer, is not guaranteed or endorsed by the publisher.
